# Effects of energy metabolism on the mechanical properties of breast cancer cells

**DOI:** 10.1038/s42003-020-01330-4

**Published:** 2020-10-20

**Authors:** Marina. L. Yubero, Priscila M. Kosaka, Álvaro San Paulo, Marcos Malumbres, Montserrat Calleja, Javier Tamayo

**Affiliations:** 1 Bionanomechanics Lab, Instituto de Micro y Nanotecnología, IMN-CNM (CSIC), Isaac Newton 8 (PTM), E-28760 Tres Cantos, Madrid Spain; 2 Cell Division and Cancer Group, Centro Nacional de Investigaciones Oncológicas (CNIO), C/ Melchor Fernández Almagro, 3, E-28029 Madrid, Spain

**Keywords:** Mechanisms of disease, Biophysics, Cytoskeleton, Breast cancer

## Abstract

Tumorigenesis induces actin cortex remodeling, which makes cancerous cells softer. Cell deformability is largely determined by myosin-driven cortical tension and actin fiber architecture at the cell cortex. However, it is still unclear what the weight of each contribution is, and how these contributions change during cancer development. Moreover, little attention has been paid to the effect of energy metabolism on this phenomenon and its reprogramming in cancer. Here, we perform precise two-dimensional mechanical phenotyping based on power-law rheology to unveil the contributions of myosin II, actin fiber architecture and energy metabolism to the deformability of healthy (MCF-10A), noninvasive cancerous (MCF-7), and metastatic (MDA-MB-231) human breast epithelial cells. Contrary to the perception that the actin cortex is a passive structure that provides mechanical resistance to the cell, we find that this is only true when the actin cortex is activated by metabolic processes. The results show marked differences in the nature of the active processes that build up cell stiffness, namely that healthy cells use ATP-driven actin polymerization whereas metastatic cells use myosin II activity. Noninvasive cancerous cells exhibit an anomalous behavior, as their stiffness is not as affected by the lack of nutrients and ATP, suggesting that energy metabolism reprogramming is used to sustain active processes at the actin cortex.

## Abstract (web summary)

Yubero et al. report a close connection between energy metabolism and cell stiffness in breast cancer cells, finding that healthy cell stiffness is based on ATP-driven actin polymerization, whereas in metastatic cells it is based on ATP-driven myosin II activity. They show that noninvasive cancerous cells exhibit an anomalous behavior, as their stiffness is little affected by the lack of nutrients and energy.

## Introduction

It is now broadly appreciated that cells can be described as mechanical systems with properties governed by biochemical cues^1,2^. The actin cortex is the main determinant of cellular mechanics^3^. This is a submembranous shell comprising a roughly isotropic polymeric network of semiflexible actin filaments (filamentous actin, or F-actin) cross-linked by specialized actin-binding proteins and containing motor proteins that generate stress within the network^4,5^. Tumorigenesis causes drastic changes in the structure and composition of cell actin cortex^3,6,7^. The main consequence is that cancerous cells become softer^7–11^, which seems to provide a key advantage for undergoing uncontrolled division, infiltration, and migration^8,12^. A major understanding of the biological significance of the mechanical switch in cancer requires deciphering the changes produced in the cytoskeleton cortex at three levels: components, organization, and activity. Unfortunately, our knowledge about these mechanisms is still poor. A reason is the difficulty to visualize the actin cortex with optical microscopies due to its location and small thickness, ∼100 nm^3,5^. In this complex puzzle, most prior work points to the apical actin network architecture and the cortical tension generated by myosin II motors, which create contractile stresses by pulling actin filaments with respect to one another^3,5,13^. The weight of each effect is often polarized in the literature. Several studies based on fluorescence microscopy and atomic force microscopy have shown that the actin network is generally less dense and less organized in cancer cells, suggesting that changes organization of the actin cortex is the main cause of the cancer cell deformability^10,14,15^. On the other hand, some reports point out that cell stiffness is dominated by cortex tension, and thus the activity of myosin II motor plays a critical role^4,5,16–18^. Surprisingly, little attention has been paid to the fact that the actomyosin cortex is sustained by active processes with characteristic times from seconds to tens of seconds that require energy in the form of ATP^3–5,19^. Cancerous cells demand higher levels of energy than their normal counterparts in order to fuel their uncontrolled cell growth and division. Anomalous energy metabolism is an essential hallmark of cancer, as discovered by Nobel Prize winner Otto Warburg in the 1920s^20–22^.

In order to identify the critical changes of the actin cortex that the cells undergo in cancer, accurate methods are needed to quantify the overall mechanical properties of individual cells. Cells are extraordinarily complex materials. Strikingly, their mechanical properties obey a simple behavior: the complex stiffness depends on frequency by a power law, i.e., $\sim \!\left( {i\omega } \right)^\beta$, where $i = \sqrt { - 1}$, *β* is the power-law exponent that in general ranges between 0 (elastic solid) and 1 (viscous liquid)^23–25^. Power-law rheology response has been observed for a broad range of diverse cell types, in different conditions, and with a wide variety of methods. Moreover, the power-law behavior extends over many decades in frequency, namely between 0.01 and 1 kHz. The origin of this mechanical response remains puzzling. A plausible explanation comes from the soft-glassy rheology theory that describes the cytoskeleton as a disordered metastable network of elements, which are held together by weak attractive forces and, as a result, trapped in energy wells with a wide range of depths. The power-law exponent *β* is related to the effective temperature of the material (amount of the agitation energy in the system), which determines the probability of elements to jump between the energy wells and reflects the system’s dynamics^26^.

The most widespread technique for measuring the mechanical properties of cells is atomic force microscopy (AFM). In this technique, the deflection of a flexible microcantilever with a nanometer or micrometer scale probe attached to its end is measured during the indentation of the cell by the probe. The resulting force (∼deflection) vs indentation curves are fitted to analytical mechanical contact models to derive the elasticity modulus of the cell^10,27–30^. Unfortunately, this approach obviates the power-law behavior of the cells that makes that the force strongly depends on the loading history. Very recently, a model that unifies contact mechanics and power-law behavior has been developed opening the door to more accurate description of the mechanical phenotype of the cells^31^.

The aim of this work is to elucidate how the actin cortex organization, the cortical tension, and cell metabolism build up the cell stiffness, and how these contributions are modified in cancer and metastasis. To this end, we measure the power-law rheology parameters of human breast epithelial cell lines with different degrees of malignancy by implementing computational methods to obtain power-law parameters from traditional AFM force vs indentation curves^31^. We get insight into the different contributions of the actin cortex to the mechanical properties of the cell by selectively disrupting actin filaments with cytochalasin D, inhibiting myosin II activity with blebbistatin, and by subjecting cells to energy starvation conditions.

## Results and discussion

### Power-law rheology analysis of cells

**Fig. 1:**
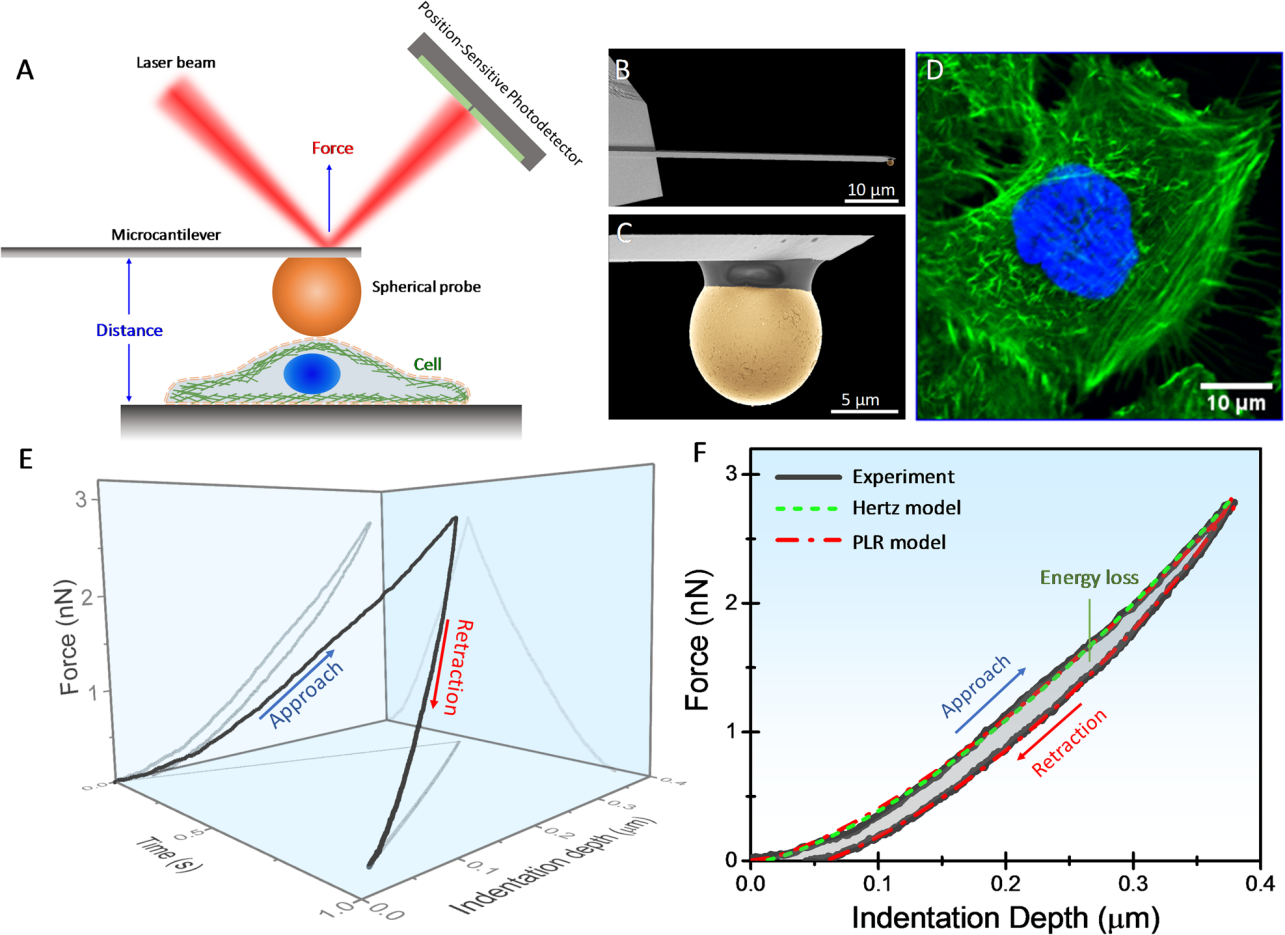
Probing the mechanical properties of living cells with atomic force microscopy. **A** Schematic illustration of the experimental set-up for measuring the force between a microsphere attached to the free end of a compliant microcantilever and the cell. The cantilever deflection is measured by the laser beam deflection method. **B** Scanning electron microscopy image of a microcantilever used in this work. The cantilevers are made of silicon and are 450 µm long, 50 µm wide and 2 µm thick. The nominal spring constant is 0.2 N/m. **C** Scanning electron microscopy image of the 10 µm diameter borosilicate glass sphere used to indent the cell. **D** Fluorescence microscopy image of a fixed MCF-10A cell that shows the actin filaments (green) and the nucleus (blue). **E** Three-dimensional curve of the force vs cell’s indentation depth and time obtained in an MCF-10A cell in normal conditions. **F** Force vs indentation depth plane of the 3D curve shown in **E**. The hysteresis between the approaching and retraction phases indicates the viscoelastic behavior of the cell that gives rise to energy loss. Hertz’s model can only fit the approaching phase. Numerical methods based on Ting’s model and power-law rheology fit the whole curve.

The cells were indented with rigid spherical probes of diameter 10 µm attached to the free end of a compliant microcantilever (Fig. 1A–D). The force is the cantilever spring constant (≈0.2 N/m) times the cantilever deflection that is measured by the laser beam deflection method. The microcantilever is approached up to reach a maximum force of about 2.5–3 nN, leading to indentation depths that range from ∼100 nm to 1–2 µm. Then the microcantilever is retracted up to reaching its original position. The force vs displacement is processed to obtain the force vs indentation depth. The large colloidal probe and the relatively high force were intended to blur out spatial heterogeneities in the three dimensions of the cells, and thus obtaining global information on the mechanical properties of the cell as a whole. Figure 1E shows the force as a function of the indentation depth and time obtained in an MCF-10A at a loading rate of 1 µm/s. The time dimension in elastic samples is meaningless, which implies that the force-indentation plane contains all the needed information to obtain the elasticity modulus. Viscoelastic behavior is manifested as a hysteresis loop in the force vs indentation depth curve (Fig. 1F). In this case, the force is not just a function of the indentation depth, it also depends on the loading history and therefore on time. Commonly, an apparent elasticity modulus is obtained by fitting the approaching phase to Hertz’s model, thus ignoring the retracting phase and therefore the sample viscoelasticity (Fig. 1F). Very recently, Efremov and Raman have developed numerical procedures based on Ting’s model that allow integrating arbitrary linear viscoelastic constitutive equations with the Hertz’s model to describe the probe-sample interaction as a function of the indentation depth and time^31,32^. We here use this approach assuming that viscoelasticity arises from the soft-glassy behavior of the cells. Thus, the elasticity relaxation modulus of the cell is given by $E_0\left( {\frac{t}{{t_0}}} \right)^\beta$, where *E*_0_ is the apparent elasticity modulus at the arbitrary reference time *t*_0_ defined here as 1 s. Figure 1F shows that numerical fitting to this model perfectly fits both the approaching and retraction phases. We notice that the adhesion forces between the probe and the investigated cells were negligible in all the conditions examined here, which is required for applying the Hertz’s model to describe the contact mechanics.

**Fig. 2:**
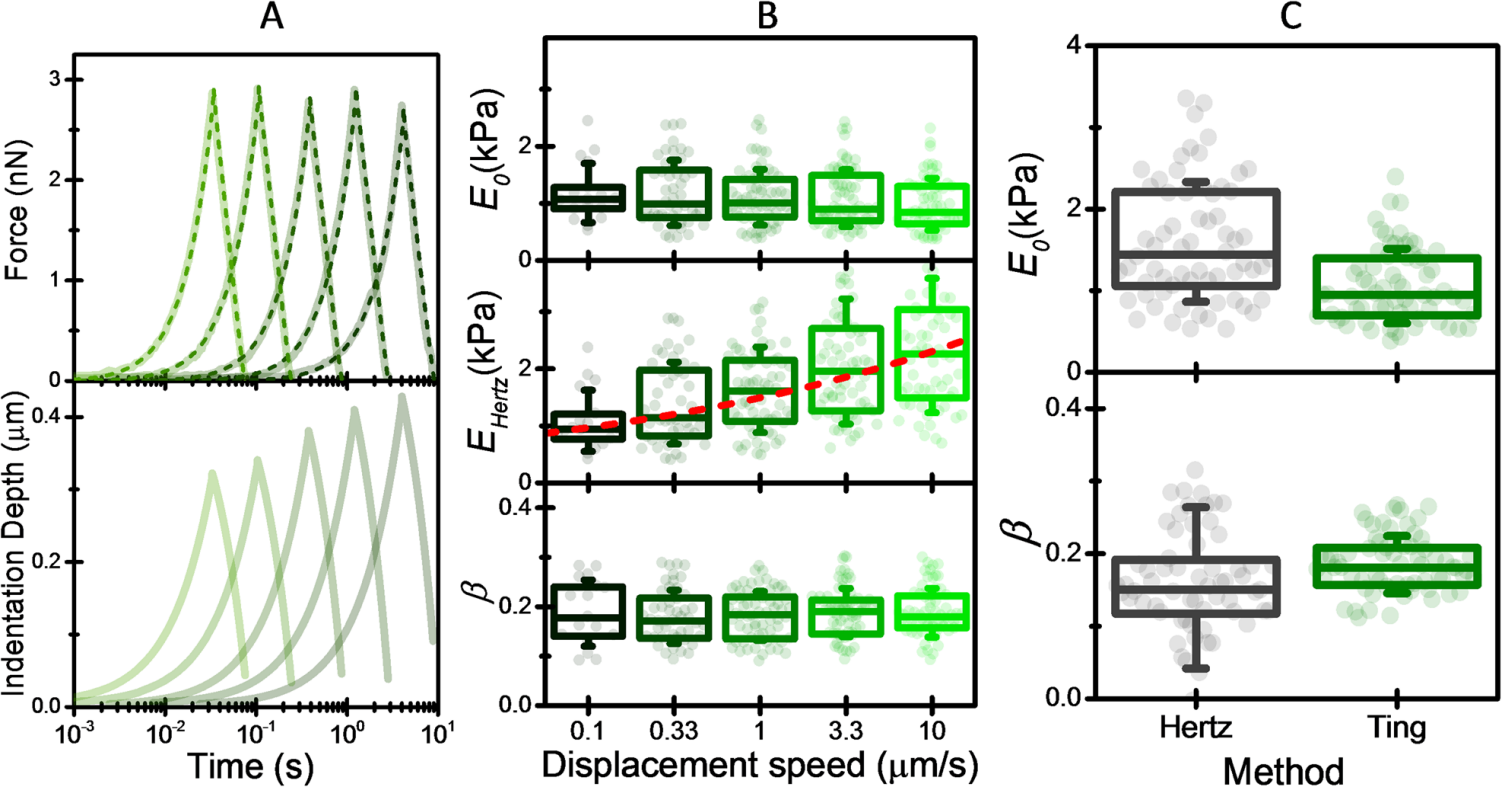
Flowchart of the method used to measure the power-law rheology parameters of the cell. **A** Force and indentation depth as a function of indentation time for loading rates of 0.1, 0.33, 1, 3.3, and 10 µm/s (thick line). Fit to the Ting’s model is also shown (dash lines). **B** Box plots of power-law rheology parameters, *E*_0_ and *β* obtained by fitting the force curves to the Ting’s model for loading rates of 0.1, 0.33, 1, 3.3, and 10 µm/s. The elasticity modulus obtained by the Hertz’s model is also shown (middle graph). The line in the Hertz elasticity modulus box represents the fit of the mean values to a power-law. Symbols represent experimental data, the box the (25%, 75%) quantiles, and the error bar the standard deviation. *n* = 17 (0.1 µm/s), *n* = 48 (0.33 µm/s), *n* = 67 (1 µm/s), *n* = 71 (3.3 µm/s), *n* = 64 (10 µm/s). **C** Comparison of the PLR parameters obtained by fitting the loading-rate dependence of the Hertz elasticity modulus with those obtained by applying the Ting’s method at each force curve and averaging over the loading rates. Symbols represent experimental data, the box the (25%, 75%) quantiles, and the error bar the standard deviation. *n* = 66 MCF-10A cells.

Figure 2A shows the force and indentation depth as a function of the time obtained in an MCF-10A cell at loading rates of 0.1, 0.33, 1, 3.3, and 10 µm/s and the numerical fitting to the PLR-Ting model. The excellent agreement between the experiment and the model clearly demonstrates that the mechanical properties of the cells can be described by the two PLR parameters, *E*_0_ and *β*. In order to validate the consistency of the methodology, we compare the PLR parameters obtained in MCF-10A cells at the different loading rates (Fig. 2B). The data show weak dependence of the power-law behavior with the loading rate, demonstrating that the method provides power-law rheology parameters that satisfactorily describe the mechanical properties of the cells in a wide range of interaction times that spans more than three logs. We notice that in previous works, the PLR parameters have been derived from the loading-rate dependence of the elasticity modulus obtained by fitting the approaching phase of the force curve to the Hertz’s model^31^. Figure 2C shows the comparison between the PLR parameters obtained by the Ting’s model (averaged over the loading rates) with those obtained by using the approach based on the Hertz’s model. Examination of the data shows that the distributions of the PLR parameters obtained by the latter approach are wider. Importantly, the power-law exponent is underestimated. In contrast to Ting’s model, the approach applied for obtaining the PLR parameters based on Hertz’s model is not theoretically supported, and thus it only provides a crude approximation to the real values. Hereinafter, the cell mechanical properties are characterized by the PLR parameters *E*_0_ and *β* obtained by the Ting’s model averaged across the five loading rates used here.

### Mechanical signatures of tumorigenesis and invasiveness

**Fig. 3:**
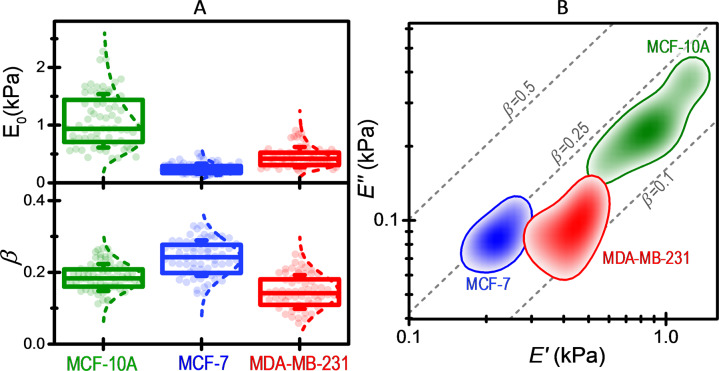
Power-law rheology parameters of the studied breast cell lines in normal conditions. **A** Box plots of the apparent elastic modulus at a reference time of 1 s, *E*_0_, and the power-law exponent, *β*. Symbols represent experimental data, the box the (25%, 75%) quantiles, and the error bar the standard deviation. The *E*_0_ values were fitted to a logarithmic normal distribution, whereas the *β* values were fitted to a normal distribution. *n* = 66 MCF-10A cells, *n* = 67 MCF-7 cell, *n* = 61 MDA-MB-231 cells. **B** Color intensity map of the probability density of the real and imaginary parts of the stiffness (*E*′, *E*″) for each cell line. The probability density contours (thick lines) represent half of the maximum probability density. Dash lines are isolines of constant *β*.

Figure 3A shows the PLR parameters of non-tumorigenic MCF-10A, tumorigenic non-metastatic MCF-7 and metastatic MDA-MB-231 cells. The results show that tumorigenic cells are softer than their healthy counterparts. In fact, *E*_0_ is 1.14 ± 0.65, 0.26 ± 0.16, 0.46 ± 0.24 kPa for the benign, malignant, and invasive phenotypes, respectively. Interestingly, invasive cancer cells are stiffer than non-invasive cancer cells. These results put into question whether the deformability of cells can be used as a biomarker of metastatic potential^33–35^. The correlation between deformability and invasiveness has been simply justified by the need of the metastatic cells of deforming through narrow gaps as they invade surrounding tissues and transit to distant sites^8,36^. However, the higher migration capability of metastatic cancer cells also requires an efficient transformation of chemical energy into mechanical work. In this mechanism, the activity of the molecular motor myosin II on its substrate actin filaments is fundamental^35,37–39^. The need for a robust actomyosin network for migration provides a plausible explanation to the higher stiffness of MDA-MB-231 cells with respect to MCF-7 cells. This hypothesis is reinforced below in the analysis of the effect of cytoskeletal drugs and ATP depletion on the cell mechanical response. The three cell lines also show important differences in the power-law exponent, *β*, that is 0.186 ± 0.049 for MCF-10A cells, 0.234 ± 0.060 for MCF-7 cells, and 0.147 ± 0.061 for MDA-MB-231 cells. Non-invasive cancer cells exhibit more fluid-like behavior than their normal counterparts. Interestingly, metastatic cells exhibit the lowest power-law exponent, suggesting that an elastic-like behavior of metastatic cells can ease enhanced migration and transiting through narrow pores.

Figure 3B shows the two-dimensional probability density of the real (*E*′) and imaginary (*E*″) parts of the stiffness, $E_0{\mathrm{cos}}\left[ {\frac{\pi }{2}\beta } \right]$ and $E_0{\mathrm{sin}}\left[ {\frac{\pi }{2}\beta } \right]$, respectively. The data shows that the three cell lines examined here exhibit three distinguishable mechanical phenotypes based on these two-orthogonal parameters. We remark that this optimal cell classification relies on the robustness and precision of the method used here for measuring the power-law mechanical response of the cells.

### Effect of cytoskeleton drugs and ATP depletion on cell rheology

**Fig. 4:**
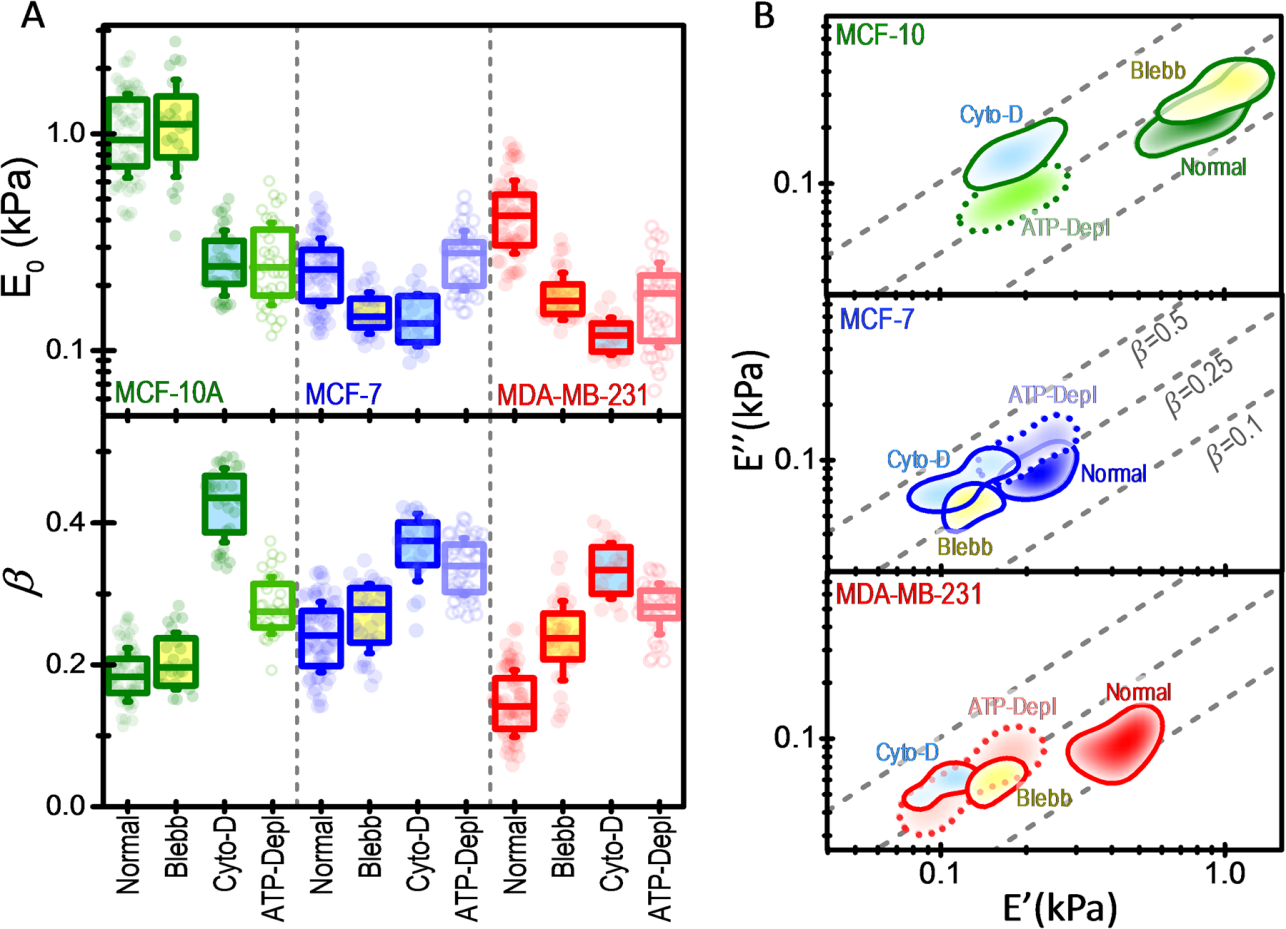
Power-law rheology parameters of the studied breast cell lines in normal conditions, treated with blebbistatin, treated with cytochalasin D, and in ATP-depletion conditions. **A** Box plots of the apparent elastic modulus at a reference time of 1 s, *E*_0_, and the power-law exponent, *β*. The symbols represent the experimental values, the box the (25%, 75%) quantiles, and the error bar the standard deviation. *n*(MCF-10A) = 66 (normal), 25 (blebb), 33 (cyto-D), 37 (ATP-Depl); *n*(MCF-7) = 67 (normal), 32 (blebb), 26 (cyto-D), 46 (ATP-Depl); *n*(MDA-MB-231) = 61 (normal), 32 (blebb), 21 (cyto-D), 37 (ATP-Depl). **B** Color intensity map of the probability density of the real and imaginary part of the stiffness (*E*′, *E*″) for each cell line and the different conditions. The probability density contours (thick lines) represent half of the maximum probability density. Dashed lines are isolines of constant *β*.

Three key components determine the mechanical properties of the cell actin cortex: the actin network organization, the actin/myosin II interaction, and energy-sustained processes such as actin polymerization/depolymerization and motor activity. These components are targeted here by treating the cells with the actin disruptor cytochalasin D, the myosin II inhibitor, blebbistatin, and by ATP depletion, respectively. Cytochalasin D is a mycotoxin that inhibits actin polymerization and disrupts actin microfilaments^40,41^. Blebbistatin blocks the myosin II in actin-detached state heads, but it does not interfere with the binding of myosin to actin nor with ATP-induced actomyosin dissociation^42,43^. Thus, blebbistatin just inhibits the transition into force-producing states. ATP depletion prevents both, motor and polymerization activity in the cell. At the level of the actin cortex, force generation by myosin II and actin polymerization are both inhibited^44,45^. Figure 4 summarizes the effects of cytoskeleton drugs and ATP depletion on the mechanical properties of the cells. Figure 4A shows separately the statistics of *E*_0_ and *β* that will be referred to as stiffness modulus and fluidity parameters hereinafter. Figure 4B shows the two-dimensional probability density of the real and imaginary parts of the stiffness. Cytochalasin D produced the most drastic changes in the mechanical properties of the cells, leading to an extraordinary enhancement of both the deformability and fluidity of the cells. The stiffness modulus dropped about four times in the normal and metastatic cells and about half in the non-invasive cancer cells. In the 2D stiffness complex space, the probability densities of untreated and cytochalasin D treated cells can be clearly separated. Disarrangement of the actin cortex makes that the cell stiffness relies on other cell mechanical components. Recent works point out to the importance of the cytoplasm, the largest part of the cell, in the mechanical resistance of the cell to deformation^46,47^. Cytoplasm rheology critically depends on the spatial and temporal scale of the deformation. In our experimental conditions, the cytoplasm response approaches to that of a viscous fluid^46,47^. This is consistent with the large increase of the fluidity observed in our experiments upon cytochalasin D treatment.

We now pay attention to the effects of ATP depletion and myosin II motor inhibition on normal and metastatic cells. ATP depletion gave rise to a decrease of the stiffness modulus of the normal cells and metastatic cells very similar to that induced by cytochalasin D. The fluidity also increased, although less than with cytochalasin D. In the stiffness complex space, the probability densities of normal cells subject to ATP depletion and treated with cytochalasin D are centered at a similar position in the real direction and separated in the imaginary direction. In the case of metastatic cells, both distributions show large overlapping due to the variability of the PLR parameters of ATP-depleted cells. Blebbistatin treatment induces different mechanical responses in MCF-10A and MDA-MB-231 cells. Healthy cells were nearly immune to treatment with blebbistatin. The stiffness modulus remained unaltered and only a slight increase of the fluidity could be appreciated. Oppositely, the stiffness modulus of metastatic cells drops in a similar amount than in ATP-depletion conditions, although the fluidity increase is smaller. Myosin II plays a fundamental role in generating forces that enable the ameboid migration capabilities of MDA-MB-231 cells^37,38^. Indeed, migration of the metastatic cells is inhibited by blebbistatin. Our data points out that myosin II activity also controls the stiffness of metastatic cells^35^. Cortical tension is regulated by myosin II by pulling actin filaments with respect to one another.

The effect of ATP depletion on non-invasive cancerous MCF-7 cells departs from the behavior observed in the other cell lines. The stiffness modulus approximately remained unaltered in energy starvation conditions. The fluidity increased, albeit by a lower amount than under the cytochalasin D treatment. The effect of blebbistatin was similar to that observed in invasive MDA-MB-231 cells, a decrease of the stiffness modulus and an increase of the fluidity, however, these changes were less accentuated.

### Contribution of active processes to the cell elasticity

**Fig. 5:**
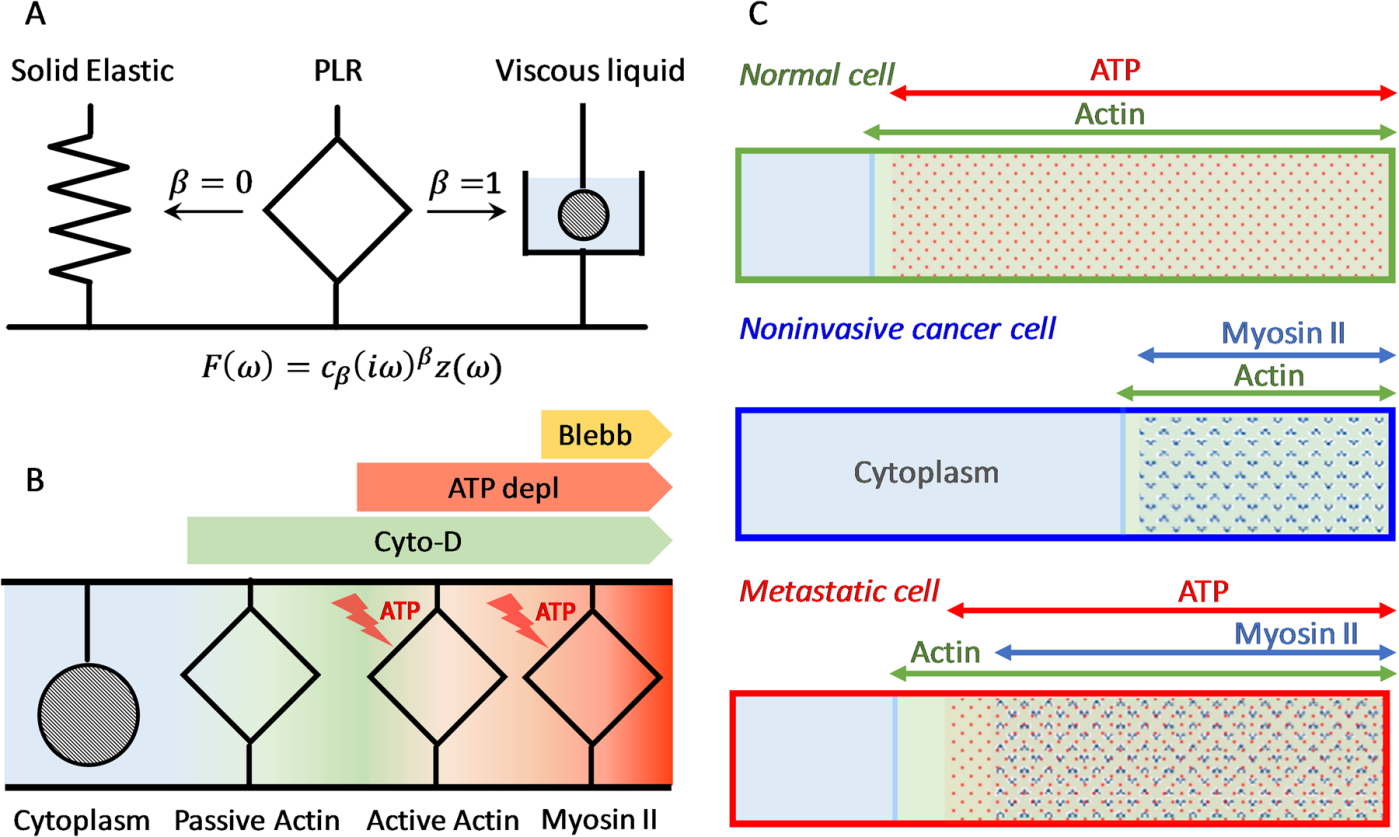
Uncoupling the effects of actin network, myosin II-driven contractility, and ATP hydrolysis on the cell stiffness. **A** Sketch of the mechanical element “springpot” used to represent PLR response. It behaves as a spring when *β* = 0 and as a dashpot when *β* = 1. **B** Schematic of the different springpots that act in parallel to provide stiffness to the cell. The model distinguishes between actin cortex components that support energy starvation (passive actin) and those that require energy consumption (active actin and myosin II). We assume fluid-like behavior for the cytoplasm in our experimental conditions (see main text). **C** Relative contributions of the cytoplasm, actin network, myosin II and ATP hydrolysis to the overall elasticity of the studied breast cell lines.

In order to obtain a major understanding of these results, we propose a simple phenomenological model that describes the mechanical response of the cell as the result of mechanical elements arranged in parallel representing the contribution of myosin II activity, the apical actin network, and the rest of cell components, mostly the cytoplasm. Each mechanical element exhibits PLR response and it is represented as “springpot” instead of the traditional combination of springs and dashpots used to describe viscoelastic materials^48^ (Fig. 5A). Importantly, the model distinguishes between actin cortex components that support energy starvation and those that require energy consumption. Although actin polymerization is a major consumer of ATP, cells account for well-conserved mechanisms to stabilize the actin cytoskeleton in absence of ATP^45,49^. This is consistent with a quiescent state of the cell, in which the actin cortex adopts a minimal but stable conformation. Confocal fluorescence microscopy shows that the actin filaments are mostly preserved in ATP-depletion conditions (Supplementary Fig. 1). A schematics of the model is shown in Fig. 5B. In our model, we assume that the contributions of the actin network, myosin II, and energy-driven actomyosin are targeted by cytochalasin D, blebbistatin, and ATP depletion, respectively. Based on this model, we represent the relative contributions of these components to the elastic component of the stiffness given by $E_0{\mathrm{cos}}\left( {\frac{\pi }{2}\beta } \right)$ (Fig. 5C).

The model indicates that the actin cortex contributes to 75–80% of the elasticity in normal and metastatic cells. In non-invasive cancerous cells, this contribution is largely reduced to about 40%. This differential behavior is consistent with the extraordinary softness of these cells, indicating that they have a minimal actin cortex configuration. In normal cells, most of the actin cortex elasticity is based on ATP-driven processes (≈95%), mostly actin polymerization processes. The contribution of myosin II activity to the cell elasticity is marginal. Similarly, in metastatic cells, ATP hydrolysis sustains most of the actin cortex elasticity (≈85%). However, most of the “active” actin cortex elasticity arises from myosin II activity (≈90%). Relevant conclusions can be drawn from the results obtained for MCF-10 and MDA-MB-231 cells. Contrarily to the vision that the actin cortex acts as a passive structure that provides mechanical resistance to the cell, this mechanical resistance is only achieved if the actin cortex is activated by ATP-driven processes. Moreover, normal and metastatic cells differ in the process in which they spend their energy. MCF-10A cell stiffness is mostly sustained by actin polymerization, whereas in the case of MDA-MB-231 cells, the stiffness largely relies on myosin II activity.

Under this perspective, non-invasive cancerous cells exhibit an anomalous metabolic effect on the elasticity. The actin cortex elasticity is insensitive to the lack of ATP and nutrients, but it drops about 75% when myosin II activity is inhibited. This surprising result suggests that MCF-7 cells can sustain motor activity in starvation conditions. The energy cost is small since MCF-7 cells exhibit an economic actomyosin cortex. We hypothesize that MCF-7 cells can use alternative routes to sustain some active processes. A common feature of cancer cell metabolism is the ability to acquire necessary nutrients from a frequently nutrient-poor environment and utilize these nutrients to both maintain viability and build new biomass^21,22^. Even in our conditions, where there are not available extracellular nutrients, some cancer cells can withstand long periods via the self-catabolic process of autophagy.

### Conclusion

Here, we demonstrate that AFM can provide precise two-dimensional mechanical phenotyping of cells based on their power-law rheology parameters. The data shows that the three cell lines examined here, breast epithelial cell lines representing normal tissue, non-invasive cancer, and metastatic cancer, exhibit three distinguishable mechanical phenotypes. The combination of accurate mechanical phenotyping with cytoskeleton drug and ATP-depletion treatments allows delineating the contributions of the apical actin network, myosin II-driven contractile tension, and ATP activity to the deformability of the cells. Relevant conclusions can be drawn from the methodology used here. These conclusions go beyond the well-known mantra “cancerous cells are softer than normal counterparts”. Contrarily to the vision that the actin cortex acts as a passive structure that provides mechanical resistance to the cell, we find that this is only true when the actin cortex is activated by metabolic processes. The results show marked differences in the nature of the active processes that build up cell stiffness. Healthy cells use ATP-driven actin polymerization, whereas metastatic cells use myosin II activity. Non-invasive cancerous cells exhibit an anomalous behavior, as their stiffness is little affected by the lack of nutrients and ATP, suggesting that energy metabolism reprogramming is used to sustain active processes at the actin cortex. In conclusion, we show the close connection between energy metabolism and cell stiffness. Remodeling of the cytoskeleton and reprogramming of energy metabolism are relevant cancer hallmarks. Our work provides a methodology to shed light on the connection between these two cancer hallmarks as well as for performing drug-assays.

## Methods

### Cell culture

MCF-7, MDA-MB-231, and MCF-10A cell lines were purchased from the American Type Culture Collection (ATCC®, USA). MCF-7 and MDA-MB-231 were grown in Dulbecco’s modified Eagle’s medium (DMEM, Gibco, Life Technologies Corporation, Rockville, MD, USA) supplemented with 10% FBS, 500 U/ml penicillin, and 0.1 mg/ml streptomycin. MCF-10A cells were cultured in DMEM/F12 medium (Gibco) supplemented with 5% horse serum, 20 ng/ml epidermal growth factor, 0.5 µg/ml hydrocortisone, 100 ng/ml cholera toxin, 10 µg/ml insulin and 500 U/ml penicillin and 0.1 mg/ml streptomycin. Cells were maintained at 37 °C in 5% CO_2_ in a humidified incubator. Since the mechanical properties depend on the intercellular contacts and cell motile state, we carried out the AFM measurement after the cells were confluent or in a steady state^50,51^. This was achieved by seeding the cells at a density of 2 × 10^5^ cells/ml onto 35 mm cell culture plates (Corning® CellBIND® Surface) after 24–36 h (Supplementary Fig. 2).

### Drug and ATP-depletion treatments

Full ATP depletion requires inhibition of oxidative and glycolytic metabolism. This was achieved by incubating the cells in glucose-free medium supplemented with 20 mM NaN_3_ and 5 mM 2-deoxy-d-glucose. The AFM measurements were carried out after 1 h of incubation. Inhibition of actin polymerization was performed by adding cytochalasin D to the cell medium up to reaching a concentration of 5 µg/ml. AFM measurements were carried out 10 min after. In order to inhibit non-muscle myosin II, blebbistatin was added to the cell medium (50 µM) for 1 h prior to the AFM measurement. Treatments were not removed while measuring with the AFM. In all the treatments, the doses and incubation times were well above the threshold for reaching the saturation response of the cells^52–55^, but keeping their viability^56–58^.

### Atomic force microscopy

AFM experiments were performed with a JPK NanoWizard^®^ 4 mounted on an inverted optical microscope (Leica DMI 6000-CS, Germany). Force-distance (ramps) curves were carried out with a 10 µm diameter borosilicate glass spherical probe attached to the free end of a silicon microcantilever with a nominal spring constant of 0.2 N/m (CP-CONT-BSG, NanoAndMore GmbH, Germany) Prior to the measurements, the spring constant and the displacement sensitivity of the photodetector were calibrated by the automatic thermal noise analysis software of the instrument. Each cell was measured on top of the nucleus at the same position at five different tip velocities: 0.1, 0.33, 1, 3.3, and 10 µm/s. Power-law rheology analysis was performed by means of custom algorithms written in Wolfram Mathematica® (Wolfram Research) based on Efremov et al.^28^ method. The resulting power-law rheology parameters of each cell at each loading rate were firstly averaged, and the resulting values were averaged across the five loading rates. The microcantilever is approached up to reach a maximum force of about 2.5–3 nN, leading to indentation depths that range from ∼100 nm to 1–2 µm. The used algorithm based on Ting’s model to obtain the rheological parameters of the cells can include bottom-effect correction models for the sample of a finite thickness^27,28^. However, we observed neither an improvement in the fitting nor significant variations in the best fitting parameters by including the finite thickness effect. Thus, this effect was not included in the numerical fittings.

### Statistics and reproducibility

The power-law rheology data shown here corresponds to: 66 MCF-10A cells in normal conditions (4 independent experiments), 37 MCF-10A cells in ATP depletion (3 independent experiments), 33 MCF-10A cells treated with cytochalasin D (3 independent experiments), 25 MCF-10A cells treated with blebbistatin (2 independent experiments); 67 MCF-7 cells in normal conditions (5 independent experiments), 46 MCF-7 cells in ATP depletion (4 independent experiments), 26 MCF-7 cells treated with cytochalasin D (4 independent experiments), 32 MCF-7 cells treated with blebbistatin (2 independent experiments); 61 MDA-MB-231 cells in normal conditions (4 independent experiments), 37 MDA-MB-231 cells in ATP depletion (3 independent experiments), 21 MDA-MB-231 cells treated with cytochalasin D (2 independent experiments), 32 MDA-MB-231 cells treated with blebbistatin (2 independent experiments). The experiments were conducted from June 2018 to February 2020. The power-law rheology parameters showed similar statistical distributions among the different independent experiments.

### Immunocytochemistry and confocal microscopy

Cover glasses were cleaned with piranha solution and coated with poly-lysine 0.1% in milli-Q water. Then, they were dried and exposed to UV light for 4 h. Cells were cultured on the covers for 24 h and they were fixed with freshly prepared 4% paraformaldehyde for 15 min at room temperature and then washed three times with PBS for 5 min each. Afterward, cells were permeabilized for 10 min with 0.25% Triton X-100 in PBS (PBST), washing with PBS three times after. Before staining, samples were blocked with 1% bovine serum albumin (BSA) in PBST for 30 min. After three rinses with PBS for 5 min each, we added phalloidin (Alexa Fluor® 488 Phalloidin, 6.6 µM, diluted 1:20) for 15 min, rinse, and added DAPI 0.5 g/ml (Cell Signalling Technology®). After rinsing one more time in PBS, we mounted the samples with ProLong® Antifade Reagent and allowed to dry 24 h at room temperature. We sealed with nail lacquer before imaging. Confocal imaging was performed with a confocal microscope Nikon A1R HD25 at the SMOC (Centro de Biología Molecular Severo Ochoa).

### Reporting summary

Further information on research design is available in the Nature Research Reporting Summary linked to this article.

## Supplementary information

Supplementary Information

Description of Additional Supplementary File

Supplementary Data 1

Reporting Summary

## Data Availability

The source data underlying Figs. 2–4 are shown in Supplementary Data 1. Any data generated or analyzed during this study that are not included in Supplementary Data 1 are available from the authors upon request.
