# Supplementary material for: Effects of energy metabolism on the mechanical properties of breast cancer cells
Source: Commun Biol. 2020 Oct 20;3:590. doi: 10.1038/s42003-020-01330-4 (PMC7576174; doi:10.1038/s42003-020-01330-4)
Supplement: Supplementary file 1 — Supplementary Information [file 42003_2020_1330_MOESM1_ESM.pdf]

Supplementary Figure 1

Confocal fluorescence images that show changes in the organization of the actin cytoskeleton of MCF10A (A-D), MCF7 (E-I) and MDAMB231 (I-M) in normal conditions (A, E, I), in ATP depletion with 20 mM NaN_3 and 5 mM 2-Deoxy-D-glucose (B, F, J), with 5µg/ml cytochalasin D (C, G, K) and 50µM blebbistatin (D, I, M). The ATP depletion and drug treatments were applied during one hour, before fixation. These treatments did not affect to cell viability in consistency with previous works[1-3]. In all the treatments, the doses and incubation times were well above the threshold for reaching the saturation response of the cells[4-7]. Bar scale of images is 20 µm. Scale bar of orthogonal slices is 3µm. F-actin was stained with phalloidin conjugated to green-fluorescent Alexa Fluor® 488 dye (Sigma Aldrich®). In general, actin filaments are well preserved in ATP depletion conditions and blebbistatin treatment, but disrupted when cytochalasin D is added. In orthogonal slices, an increase in the height of the cells is observed with both ATP depletion and cytochalasin D additions, but not in presence of blebbistatin in the medium of the cells, although changes in cell surface size are observed.

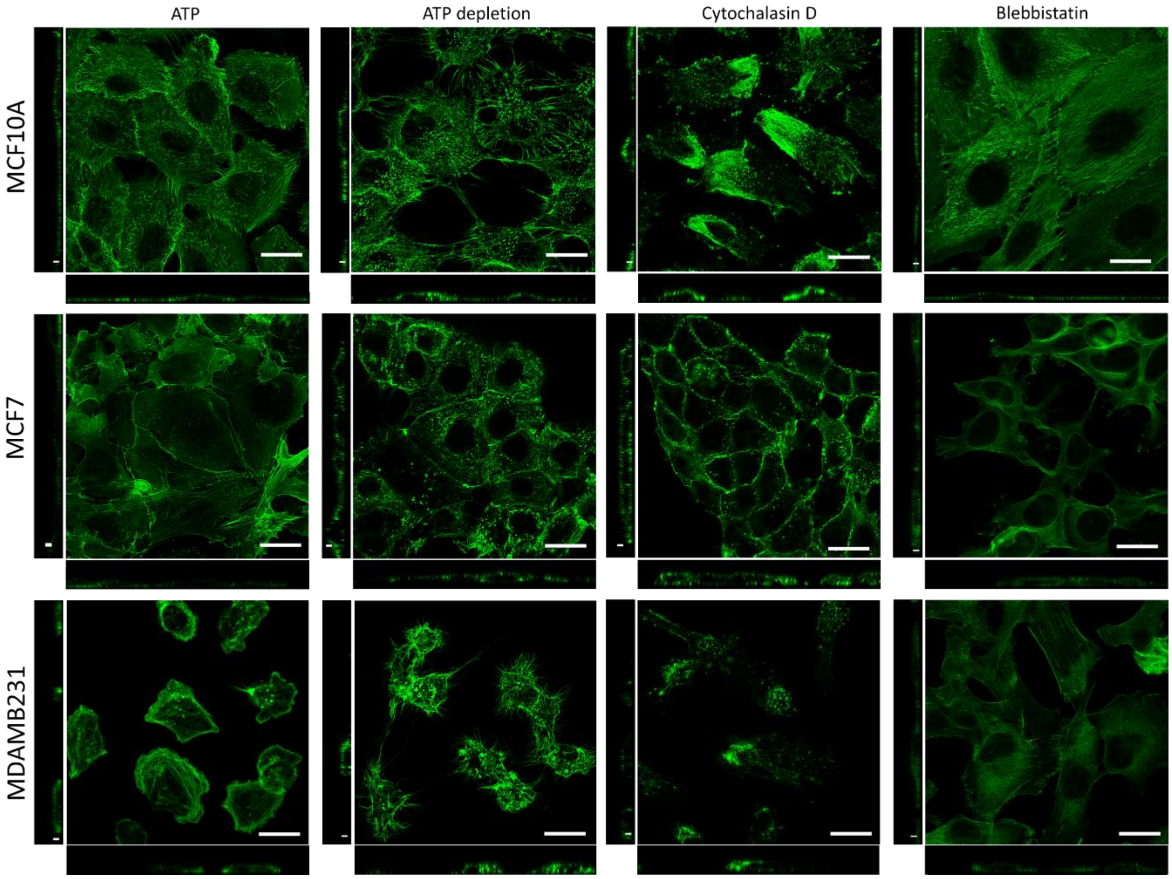

Supplementary Figure 2.

Representative bright-field optical microscopy images of MCF-10A, MCF-7 and MDA-MB-231 cells in normal conditions at the AFM measurement time. Shadow at the left is the AFM microcantilever, bright spot is the laser placed on the back of the cantilever. Scale bar is 50µm. Cells were maintained at 37º C in 5% CO_2 in a humidifed incubator. Since the mechanical properties depends on the intercellular contacts and cell motile state, we carried out the AFM measurement after the cells were confluent or in a steady state[8,9]. This was achieved by seeding the cells at a density of 2 x 10^5 cells/ml onto 35mm cell culture plates (Corning® CellBIND® Surface) after 24-36 h. MCF-10A and MCF-7 form continuous monolayers with well-defined cell borders, while MDA-MB-231 cells are isolated or in small clusters of variable cell density. MCF-10A stiffness depends on cell confluence, while MCF-7 and MDA-MB-231 do not[9]. Images were taken using a long free working distance HC PL Fluotar 10x/0,32 objective (Leica).

.

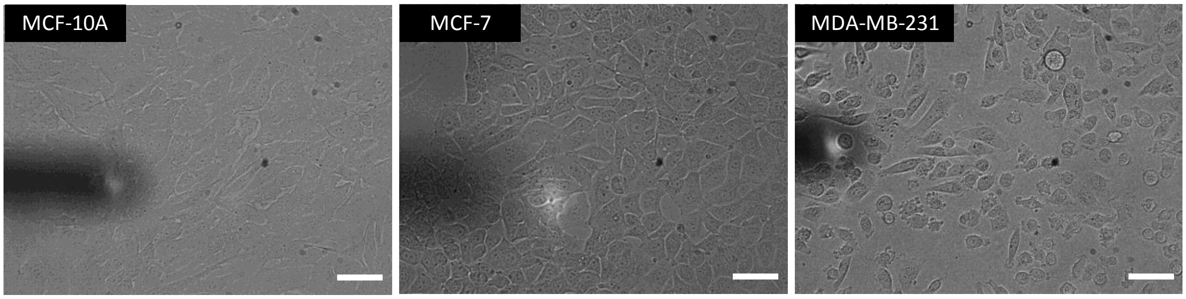

References

1 Duxbury, M. S., Ashley, S. W. & Whang, E. E. Inhibition of pancreatic adenocarcinoma cellular invasiveness by blebbistatin: a novel myosin II inhibitor. *Biochemical and biophysical research communications* **313**, 992-997 (2004).

2 Hayot, C. *et al.* Characterization of the activities of actin-affecting drugs on tumor cell migration. *Toxicology and applied pharmacology* **211**, 30-40 (2006).

3 Bizjak, M. *et al.* Combined treatment with Metformin and 2-deoxy glucose induces detachment of viable MDA-MB-231 breast cancer cells in vitro. *Scientific reports* **7**, 1-14 (2017).

4 Straight, A. F. *et al.* Dissecting temporal and spatial control of cytokinesis with a myosin II Inhibitor. *Science* **299**, 1743-1747 (2003).

5 Pogoda, K. *et al.* Depth-sensing analysis of cytoskeleton organization based on AFM data. *European Biophysics Journal* **41**, 79-87 (2012).

6 Ayala, Y. A. *et al.* Effects of cytoskeletal drugs on actin cortex elasticity. *Experimental cell research* **351**, 173-181 (2017).

7 Wakatsuki, T., Schwab, B., Thompson, N. C. & Elson, E. L. Effects of cytochalasin D and latrunculin B on mechanical properties of cells. *Journal of cell science* **114**, 1025-1036 (2001).

8 Efremov, Y. M. *et al.* The effects of confluency on cell mechanical properties. *Journal of biomechanics* **46**, 1081-1087 (2013).

9 Schierbaum, N., Rheinlaender, J. & Schäffer, T. E. Viscoelastic properties of normal and cancerous human breast cells are affected differently by contact to adjacent cells. *Acta biomaterialia* **55**, 239-248 (2017).
