# Supplementary material for: Effects of energy metabolism on the mechanical properties of breast cancer cells
Source: Commun Biol. 2020 Oct 20;3:590. doi: 10.1038/s42003-020-01330-4 (PMC7576174; doi:10.1038/s42003-020-01330-4)
Supplement: Supplementary file 2 — Description of Additional Supplementary File [file 42003_2020_1330_MOESM2_ESM.pdf]

Description of additional supplementary file

File name: Supplementary Data 1. Source data for main figures 2 – 4.
